# Intestinal Permeability, Dysbiosis, Inflammation and Enteric Glia Cells: The Intestinal Etiology of Parkinson’s Disease

**DOI:** 10.14336/AD.2022.01281

**Published:** 2022-10-01

**Authors:** Huijia Yang, Song Li, Weidong Le

**Affiliations:** ^1^Center for Clinical Research on Neurological Diseases, the First Affiliated Hospital, Dalian Medical University, Dalian, China.; ^2^Liaoning Provincial Key Laboratory for Research on the Pathogenic Mechanisms of Neurological Diseases, the First Affiliated Hospital, Dalian Medical University, Dalian, China.; ^3^Department of Neurology and Institute of Neurology, Sichuan Academy of Medical Science-Sichuan Provincial Hospital, Chengdu, China.

**Keywords:** Parkinson’s disease, intestinal permeability, dysbiosis, inflammation, enteric glial cells

## Abstract

The scientific and medical communities are becoming more aware of the substantial relationship between the function of the central nervous system (CNS) and the state of the gut environment. Parkinson's disease (PD) is a neurodegenerative disorder that affects the nigrostriatal pathway in the midbrain, presenting not only motor symptoms but also various non-motor manifestations, including neuropsychiatric symptoms and gastrointestinal (GI) symptoms. Over time, our knowledge of PD has progressed from the detection of midbrain dopaminergic deficits to the identification of a multifaceted disease with a variety of central and peripheral manifestations, with increased attention to the intestinal tract. Accumulating evidence has revealed that intestinal disorders are not only the peripheral consequence of PD pathogenesis, but also the possible pathological initiator decades before it progresses to the CNS. Here, we summarized recent research findings on the involvement of the intestinal environment in PD, with an emphasis on the involvement of the intestinal barrier, microbiome and its metabolites, inflammation, and enteric glial cells

Parkinson's disease (PD) is the second most common neurodegenerative disorder and is dominated by motor symptoms. The most common pathological signature in the PD brain is the intracellular inclusions containing misfolded and aggregated alpha-synuclein (α-syn), also known as Lewy bodies, resulting in the loss of dopaminergic neurons in the substantia nigra. An estimated 25-80% of PD individuals may have gastrointestinal (GI) issues, including constipation, nausea, dyspepsia, dysphagia, and excessive drooling [1, 2]. Constipation is the most frequent GI symptom in PD. Indeed, GI dysfunctions is one of the most serious non-motor symptoms of PD, with up to 30% of patients suffering from it prior to the start of central nervous system (CNS) symptoms [3]. It is extremely likely that the GI tract is a key location and origin of pathological change in the CNS of PD.

## Increased Intestinal Permeability Contribute to Parkinson's Disease

The GI tract serves as a semipermeable barrier, allowing nutrients, ions, and water to be absorbed while also regulating host interaction with a vast range of food antigens and bacterial metabolites. Therefore, intestinal leakage in PD patients may be a critical early trigger leading to the onset and/or development of PD process. Lewy bodies appear in the enteric nervous system (ENS) prior to the CNS [4, 5]. Braak was the first to hypothesize that pathogens could breach the intestinal epithelial barrier (IEB) and cause aggregated α-syn in the ENS, which could then spread to the CNS via the vagus nerve [6, 7]. In PD mouse models [8] and PD patient samples [9], an association between intestinal epithelial permeability and aggregated α-syn has been proposed.

**Figure 1.**
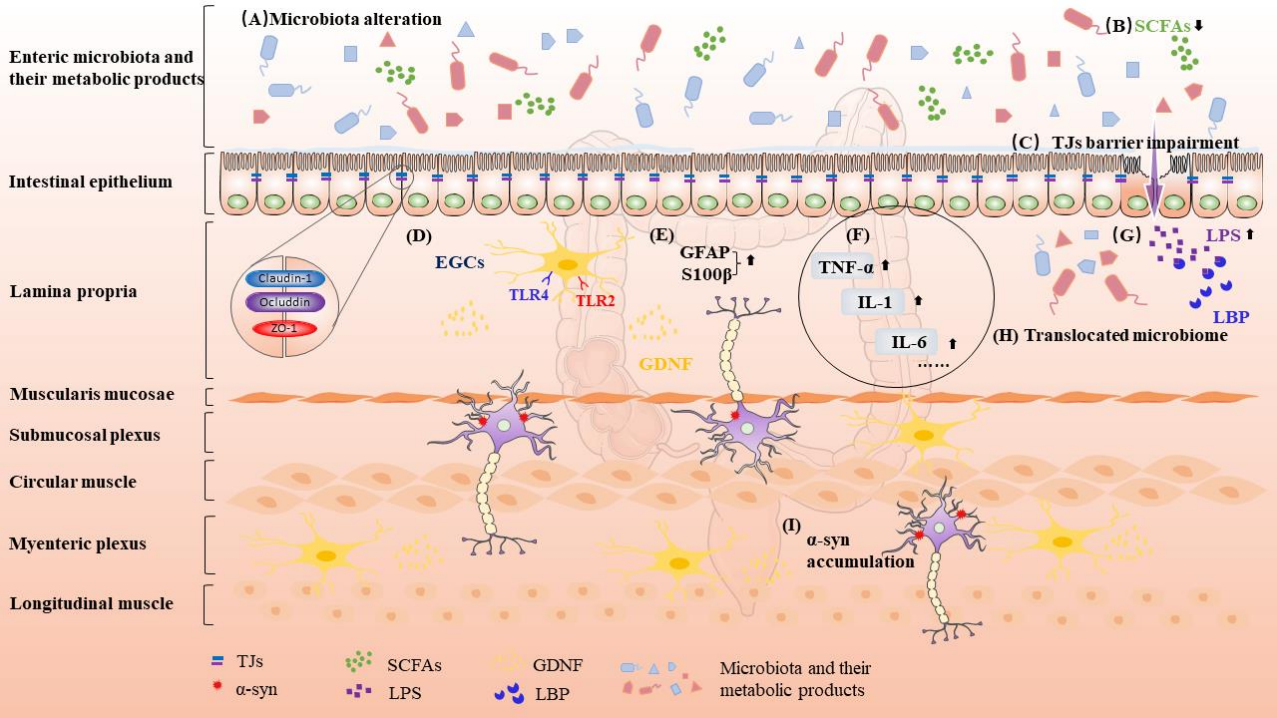
**Intestinal pathological features of PD**. (**A**) Microbiota alterations including the increased pathogens and decreased probiotics levels; (**B**) Decreased levels of SCFAs; (**C**) TJs barrier impairment leads to an increased intestinal permeability; (**D**) EGCs priming through TLRs activation; (**E**) Considerable rise of glial marker expression, such as GFAP and S100β; (**F**) Release of inflammatory cytokines; (**G**) Increased level of LPS; (**H**) Gut dysbiosis and leaky IEB promote bacterial or LPS translocation; (**I**) Increased expression and aggregation of α-syn. Abbreviation: PD, Parkinson's disease; SCFA, primarily short chain fatty acid; TJs, tight junctions; EGCs, enteric glial cells; TLR, toll-like receptor; GFAP, glial fibrillary acidic protein; LPS, lipopolysaccharide; IEB, intestinal epithelial barrier; α-syn, alpha-synuclein; TNF, tumor necrosis factor; IL, interleukin.

The intestinal barrier consists of intestinal microbiota and the outer mucous layer, as well as the epithelium and lamina propria [10]. The intestinal epithelium forms a regulated barrier between blood circulation and intestinal contents. It inhibits the transit of toxic substances and promotes nutritional absorption and excretion [11]. This barrier can be penetrated through transcellular and/or paracellular pathways [11]. Tight junctions (TJs), also called zona occludens, play an important part in intestinal barrier function, and the dysfunction of TJs may lead to increased paracellular permeability in the intestinal epithelium (Fig. 1) [12]. TJs are generated by transmembrane proteins like claudins and occluding, which are coupled to the actin cytoskeleton through high molecular weight proteins known as zona occludens [12]. Zonula controls intestinal permeability by regulating the activity of TJs. Increased zonula concentrations have been correlated with GI barrier breakdown.

The loss of TJ protein increases intestinal permeability in the context of intestinal inflammation, according to the prevalent theory of "leaky gut" [13]. (Fig. 1). For the first time, Clairembault et al. showed morphological abnormalities of IEB in patients with PD [14]. They observed the decreased occludin expression in PD individuals was not paralleled by alterations of paracellular permeability. There are two possible reasons for this, one is that occludin is not necessary to produce TJs or maintain the barrier function. Early research found no substantial abnormalities in the structure or function of intestinal TJs in occludin knockout mice [15]. Another possible explanation is that the molecular weight of fluorescein-5,6-sulfonic acid employed in the research to assess paracellular permeability is only 400 Da, which may be too small to detect IEB permeability defects caused by downregulation of occluding [14].

To date, studies attempting to assess intestinal permeability in PD patients have yielded only initial and contradictory findings. Davie et al.[16] first discovered changes in intestinal permeability in PD patients. They found that PD patients had decreased mannitol absorption, leading to an elevated lactulose/mannitol ratio, which has been used to evaluate small-intestinal permeability. In contrast, one study [17] found a slight increase in the lactulose/mannitol ratio in three of twelve PD subjects. Another study also employed sucralose absorption to assess intestinal permeability in nine PD individuals and reported no difference in lactulose/mannitol ratio between PD subjects and the control group, but PD sufferers had considerably higher colon permeability [9]. Clairembault et al. also reported no significant difference in intestinal permeability between PD subjects and healthy individuals [14]. There are three possible reasons that led to these inconsistent results. One possibility is that different assessment methods yielded inconsistent results. Some studies measured intestinal permeability by analyzing the concentration of sugar probes in urine. This noninvasive method necessitates a high level of patient cooperation. Participants were instructed to avoid consuming anything that contained similar probe sugars[18]. Other studies used endoscopic biopsies in Ussing chambers, which is an invasive method for evaluating mucosal permeability [19]. One possibility is that their sample sizes were small, and the confidence intervals were wide. Aside from analysis methods and sample size, the different disease stages of the patients included in the studies should be considered as a factor causing results inconsistency. The symptoms leading to the diagnosis of PD usually begun at 6 to 171 months before their participation in the studies [14, 16, 17].

Indeed, PD is associated with increased intestinal permeability, which may be considered a contributing factor to the development of pathology. The alteration of intestinal permeability in the context of disease may be attribute to the microenvironmental regulation of intestinal barrier function, particularly ENS.

## Involvement of Intestinal Microbiota and Their Metabolites in Parkinson’s Disease

Changes in intestinal microbiota, of course, may play a significant role in the altered intestinal permeability of PD patients, as there is convincing evidence indicating the interactions between intestinal microbiota and intestinal epithelial cells (IECs) (Fig. 1). The ratio of symbiotic to pathogenic bacteria can affect the integrity of the intestinal barrier [20]. Several studies have found that the abundance of Faecalibacterium Prausnitzii and Prevotellaceae in PD patients were reduced [20, 21]. These microbial alters could play a role in local inflammatory changes. In recent study, Dwyer et al. [22] discovered that dextran sodium sulphate (DSS)-induced (DSS, a toxin commonly used to model colitis) variations in microbiota were associated with an augmented inflammatory profile. Faecalibacterium Prausnitzii was found to have anti-inflammatory and protective effects on the IEB [23, 24]. As a result, a reduction of Faecalibacterium prausnitzii and an increase of Enterobacteriaceae may undermine the IEB, making the ENS more vulnerable to intraluminal pathogens. Most studies found lower Prevotellaceae populations in PD patients [25-27]. This genus is able to be producing short chain fatty acids (SCFAs), as well as being linked to immune cell activation and the production of pro-inflammatory proteins. In addition, Prevotella reduction may be connected with decreased synthesis of mucin, which is related to increased intestinal permeability [9]. Many studies have discovered that PD mice also have intestinal microbial dysbiosis [28]. Furthermore, they discovered that fecal microbiota transplantation (FMT) alleviates GI and motor symptoms by reducing intestinal microbial metabolic disorders [28, 29]. Although only a few cases have been reported, FMT has been shown to have therapeutic promise in PD patients [30].

Bacterial components including lipopolysaccharide (LPS) or amyloid protein curli have been demonstrated to increase α-syn aggregation, lending credence to the idea that the gut microbiome is involved in PD [31]. Kelly et al [8]. found that low-dose LPS treatment gradually increased α-syn expression, followed by intestinal leakiness, and that these increases were predominantly confined to the large intestine of mice. Escherichia coli (E. coli) and other Gram-negative bacteria produce curly-fibers. Curli-fibers are functional amyloid fibers that act as a protein scaffold in the extracellular matrix (ECM) of biofilms, accounting for 85 percent of the ECM of biofilm [32]. Curli is capable of improving intestinal barrier function. Curli-fibers directly stimulate epithelial cells, resulting in barrier strengthening and a decrease in bacterial translocation [33].

Intestinal bacteria and their metabolites, primarily SCFAs, contribute to preserving the integrity of the IEB through modulating cell proliferation and differentiation, TJ protein expression, and mucosal permeability [34]. In the colon, intestinal microbial fermentation of undigested dietary carbohydrates produces SCFAs including acetate, propionate, butyrate, and valerate. Butyrate, as the primary energy source for colon cells, is important to maintain the intestinal barrier [35]. Changes in butyrate concentration may impact the expression of occludin, a component of TJs protein, which may influence intestinal permeability. Butyrate has anti-inflammatory properties by activating SCFA receptors, resulting in anti-inflammatory, anti-microbial, and reducing the intestinal leakiness [35]. In inflammatory bowel disease (IBD), a lack of butyrate leads to TJs lesions and, eventually increased intestinal permeability [36]. Butyrate treatment improves transepithelial resistance in a Dextran Sulfate Sodium-induced colitis rat model, which is related to maintaining tight junction integrity and inhibiting tumor necrosis factor (TNF)-α release [37]. Butyrate can protect cells from increased paracellular permeability and epithelial barrier destruction caused by LPS, increase the expression of tight junction claudins and reduce the expression of inflammatory cytokines [38-41]. There is compelling evidence that SCFAs, particularly butyrate, are beneficial in various animal models of PD [42, 43]. However, only animal trials were carried out. Extensive clinical studies are required to further assess the role of SCFAs in PD.

In addition, the intestinal epithelium, being a mucosal tissue, continuously generates mucus and is covered with mucus, which serves as the first line of protection against pathogens. Mucus is generated by goblet cells and is made up of glycosylated mucin proteins as well as other protective compounds that aid in epithelial restoration. Microbiota products influence the production of intestinal mucus. Butyrate generated by benign microbiota components stimulates greater mucin secretion, forming a positive feedback loop for the preservation of the mucous barrier and its colonization by butyrate-producing commensals [44].

Reduced levels of SCFAs in the colon may impede colonic motility, resulting in constipation in PD (Fig. 1). In a study of plasma SCFAs in PD patients, researchers discovered that PD patients have higher plasma SCFAs level, which may be due to epithelial barrier damage induced by gut dysbiosis with low-grade inflammation in PD patients [45]. These studies illustrate that intestinal dysbiosis in PD patients is linked with a significantly decreased SCFAs level, resulting in impaired IEB, promoted inflammatory responses, disrupted intestinal neuronal networks, and intestinal motility dysregulation [20]. In the largest PD microbiome study to this day, Wallen et al. discovered PD patients may contain excessive opportunistic pathogens, decreased levels of SCFA-producing bacteria, and/or increased amounts of carbohydrate metabolites (commonly referred to as probiotics) in their gut microbiomes [46]. A study including 24 PD subjects and 14 controls found that the proportion of endotoxin-producing bacteria increased while the number of SCFA-producing bacteria decreased [47].

## Intestinal Inflammation May Promote Progression of Synucleinopathy from the Intestinal Nervous System to the Central Nervous System

TJ protein synthesis is influenced by intestinal bacteria, and several pro-inflammatory cytokines generated by activated immune cells act on TJs to increase barrier permeability [48]. Inflammatory circumstances generally cause intestinal bacteria to become more pathogenic and less symbiotic, thereby aggravating inflammation and raising the possibility of sustained immunological reactions in the gut [49]. In a cohort study, Villumsen et al. [50] found individuals troubled with IBD are more susceptible to PD. Inflammation is a well-known feature of PD [51]. It has been proposed that this inflammation may be triggered by a disruption in the intestinal barrier, which exposes the system to inflammatory microbial compounds such LPS, a component of bacterial cell walls [52]. The decrease of LPS-binding protein (LBP) [9, 26] and the increase of LPS [53] in plasma from PD sufferers indicate that peripheral blood tissues were more exposed to LPS, which means the presence of intestinal barrier failure. In individuals with PD, intestinal barrier malfunction and increased intestinal permeability induced by inflammation create a favorable environment for the exposure of the ENS to microbiota and their toxic products [54]. Indeed, changes in the intestinal microbiota can cause IEB breakdown and increased mucosal permeability, resulting in bacterial translocation into the mucosa and possibly systemic inflammation [55]. Changes in the intestinal microbiota metabolic, including SCFA and peptidoglycans, may further increase intestinal permeability, induce widespread neuro-inflammation, thus contributing to the development of PD [56]. Intestinal dysbiosis or intestinal leakage induced by the pro-inflammatory intestinal environment can initiate or exacerbate PD.

Forsyth and colleagues [57] discovered increased colon permeability in individuals with early PD and verified that it is linked to intestinal endotoxin exposure, oxidative stress, and α-syn aggregation. Schwiertz et al. [58] found that calprotectin, α-1-antitrypsin (A-1-AT), as well as banded protein were significantly increased in PD patients. A-1-AT is a protease inhibitor that reflects protein loss in the intestinal lumen, which may be caused by a breakdown of the mucosal barrier. Calprotectin is a member of the S100 family protein that is generated after the activation of neutrophil. Calprotectin is resistant to enzyme breakdown, making it a sensitive fecal marker for intestinal inflammation. Recently, Mulak et al. [59] also found elevated fecal calprotectin in PD patients. Aho et al. [60] observed no evidence of increased gut permeability in PD patients evaluated by stool zonulin or plasma LBP. However, their findings supported the existence of SCFA deficits and higher amounts of fecal calprotectin in PD.

## Role of Enteric Glial Cells in the Intestinal Origin of Parkinson’s Disease

Enteric glial cells (EGCs) are a unique type of peripheral glial cell that are distributed in the intermuscular, submucosal plexus, and extra-ganglionic regions like the muscular layer and mucosal lamina propria [61]. EGCs have traditionally been assumed to participate mainly to the construction and nutritional maintenance of intestinal neurons. Besides their regular roles, they are also critical for the dynamic homeostasis modulation of various GI activities, including intestinal motility and epithelial integrity, through complex interactions with neurons, immune cells and IECs [61]. Depending on where they are, EGCs have distinct physiological roles according to their distribution [62]. EGCs are found beneath the IECs and like their CNS counterparts, have a direct impact on IECs and IEB function [63], whereas EGCs in the submucosal or myenteric plexus embed neurons and modulate neurotransmission [64]. EGCs regulate epithelial barrier activities by suppressing IECs proliferation and decreasing epithelial permeability via glial-derived factors [65, 66]. The IEB integrity of mice with enteric glial ablation is dramatically altered [67]. According to a recent study, EGCs dysfunction in PD patients may play an indispensable part in adjusting to the increased intestinal permeability [10].

Many researchers have found that EGCs respond to damage activation primarily via the Toll-like receptors-2 (TLR-2) and Toll-like receptors-4 (TLR-4) [68]. TLR-4 knockout mice were partially protected from rotenone-induced PD, indicating that parkinsonian symptoms can be avoided by suppressing the EGC-mediated immunological response [54]. The discovery that EGCs not only express TLR but also distinguish between pathogens and probiotics through modifying TLR expression, highlighting the potential role of EGCs in initiating innate immune responses [68]. The intestinal microbiota dose, in fact, influences the primary colonisation of EGCs in the intestinal mucosa [69]. Although it has not been proven that certain strains are directly responsible for the onset of PD, gut-brain axis disorders caused by immune initiation of EGCs via altered intestinal microbiota is becoming more established. EGCs may function as enterotoxin entry points into the CNS, particularly in neurodegenerative disorders [70].

David et al. hypothesized that altered expression of glial markers and pro-inflammatory cytokines in the colon of PD individuals can regulate the integrity and enhance the permeability of IEB. Although glial heterogeneity has been confirmed in the intestine, there are various physiological glial markers, including Sox-10, S100β and glial fibrillary acidic protein (GFAP), which fluctuate dynamically depending on the condition of the intestinal mucosa [61]. The expression of GFAP and S100β increases dramatically in EGCs activated by cellular danger signals. This overexpression is linked to the neuroinflammatory response in the ENS mediated by EGCs [71]. EGCs release glial-derived neurotrophic factor (GDNF), which appears to be important in preserving mucosal integrity. According to several studies, EGC-derived GDNF enhances TJs in IECs and may protect IECs from cytokine-induced apoptosis [72-74]. Its preventive action is explained by the fact that it simultaneously suppresses EGC apoptosis and lowers pro-inflammatory cytokine production [75, 76]. Intestinal inflammation is intimately linked to glial dysfunction in PD, and evidence shows that EGCs is essential for controlling intestinal inflammation [77]. In addition to modulating IEB resistance, EGCs have been shown to improve IEB repair after mechanical or inflammatory damage [78].

EGC-driven neuroinflammation has been postulated as the cause of synaptic dysfunction and intestinal motility abnormalities at the early stage of PD progression since these EGCs regulate motility, intestinal permeability, and immune responses [61]. This assumption is supported by an increase in enteroglia-associated pro-inflammatory indicators in the intestinal mucosa of PD subjects, including interleukin (IL)-6, IL-1, TNF-α, GFAP, and S100β [79]. When PD colon samples were compared to control samples, Thomas et al. [80] discovered differences in intestinal GFAP expression and phosphorylation. EGCs dysfunction occurs at the onset of PD, leading to local inflammation that can extend to the CNS. This may result in impaired intestinal permeability and initial α-syn aggregation in the ENS [58].

In addition, the "pathological loop" between glial activated by aggregated α-syn and the misfolded α-syn generated by intestinal glial activation emphasizes the significance of EGCs in the pathogenesis of PD [77]. This reactive gliosis may exacerbate damage to the integrity of the IEB, resulting in neuroinflammatory response and thus accelerating the progression of the PD pathogenic process [81, 82].

## Conclusion and perspective

**Figure 2.**
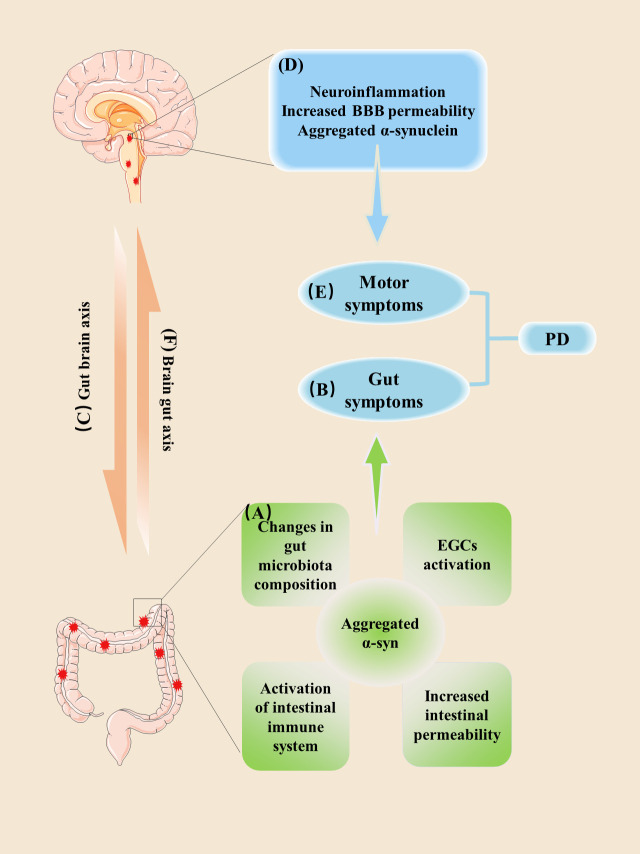
**The vicious circle between intestinal disorders and central nervous degeneration in PD**. (**A**) Increased intestinal permeability along with alterations in gut microbiota composition, and EGC activation, may result in an inflammatory response that further promotes intestinal leakiness, and increases α-syn expression and aggregation in ENS. IECs fail to fully repair the barrier, leading to a vicious cycle of barrier leakage, microbial dysregulation, chronic inflammation, and EGCs activation; (**B**) In these conditions, gut symptoms can be detected as premonitory symptoms in PD patients; (**C**) α-Syn may be transmitted from the gut to the brain via the vagus nerve; (**D**) Chronic inflammation and intestinal leakiness contribute to systemic inflammation, which can increase BBB permeability. Intestinal inflammation, systemic inflammation, and α-syn in the brain all contribute to neuroinflammation, which result in the neurodegeneration that is characteristic of PD. (**E**) Accumulation of α-syn, in the nigrostriatal area, may thus predispose parkinsonian neurodegeneration and the development of motor symptoms. (**F**) Central neuroinflammation and subsequent neurodegeneration could aggravate the enteric pathologic changes via brain-gut descending pathways, creating a positive feedback loop that may contribute to the neurodegenerative process. Abbreviation: α-syn, alpha-synuclein; EGC, enteric glial cell; ENS, enteric nervous system; IEC, intestinal epithelial cell; BBB, blood brain barrier; PD, Parkinson's disease.

Taken together, these clinical and preclinical findings from patients and various animal models suggest that increased intestinal permeability, altered intestinal microbiota, intestinal inflammation, and EGC activation are early events of the disease that occur prior to the onset of CNS symptoms (Fig. 2). At an early stage, PD patients have anomalous interplays among intestinal microbiota, intestinal inflammatory, intestinal barrier, and EGC activation, which may promote aggregated α-syn in ENS. In later stages of disease, intestinal inflammatory activation may trigger inflammatory events in the CNS via the gut-brain ascending pathway, promoting the accumulation of α-syn in the CNS. Central neuroinflammation and subsequent neurodegeneration could exacerbate enteric pathologic changes via brain-gut descending pathways, resulting in a bidirectional relationship that could contribute to the neurodegenerative process.

As the population ages, the morbidity and mortality of PD are increasing year by year globally. Oral levodopa or a dopamine agonist is still the first-line treatment for patients with PD. Intestinal dysfunction may not only be the origin of PD but also affect the absorption of drugs in the gut, accelerating the progression of the PD. Current knowledge may aid in the development of novel therapeutic interventions targeting gut functions in the early stages of the disease. Developing therapies to minimize gut dysfunction might potentially slow or halt neurodegeneration ascending to the CNS. While more studies are needed to validate the exact role of enteric pathologic in the onset of PD. Fortunately, the development of experimental models enables us to gain a better understanding of the causality and relationship between intestinal dysbiosis, permeability changes, EGCs and intestinal inflammation in PD. Meanwhile, prospective longitudinal studies in subjects at risk for PD are needed to identify which factors act as triggers to cause or promote the disease. Changes in intestinal function may become a sensitive marker for predicting and assessing the risk of PD in the future.

## References

[1] Sung HY, Park JW, Kim JS (2014). The frequency and severity of gastrointestinal symptoms in patients with early Parkinson's disease. J Mov Disord, 7:7-12. 24926404 10.14802/jmd.14002 PMC4051727

[2] Ivan IF, Irincu VL, Diaconu S, Falup-Pecurariu O, Ciopleias B, Falup-Pecurariu C (2021). Gastro-intestinal dysfunctions in Parkinson's disease (Review). Exp Ther Med, 22:1083. 34447476 10.3892/etm.2021.10517 PMC8355716

[3] Jost WH (2010). Gastrointestinal dysfunction in Parkinson's Disease. J Neurol Sci, 289:69-73. 19717168 10.1016/j.jns.2009.08.020

[4] Hilton D, Stephens M, Kirk L, Edwards P, Potter R, Zajicek J, et al. (2014). Accumulation of alpha-synuclein in the bowel of patients in the pre-clinical phase of Parkinson's disease. Acta Neuropathol, 127:235-241. 24240814 10.1007/s00401-013-1214-6

[5] Shannon KM, Keshavarzian A, Dodiya HB, Jakate S, Kordower JH (2012). Is alpha-synuclein in the colon a biomarker for premotor Parkinson's disease? Evidence from 3 cases. Mov Disord, 27:716-719. 22550057 10.1002/mds.25020

[6] Braak H, Rub U, Gai WP, Del Tredici K (2003). Idiopathic Parkinson's disease: possible routes by which vulnerable neuronal types may be subject to neuroinvasion by an unknown pathogen. J Neural Transm (Vienna), 110:517-536. 12721813 10.1007/s00702-002-0808-2

[7] Braak H, de Vos RA, Bohl J, Del Tredici K (2006). Gastric alpha-synuclein immunoreactive inclusions in Meissner's and Auerbach's plexuses in cases staged for Parkinson's disease-related brain pathology. Neurosci Lett, 396:67-72. 16330147 10.1016/j.neulet.2005.11.012

[8] Kelly LP, Carvey PM, Keshavarzian A, Shannon KM, Shaikh M, Bakay RA, et al. (2014). Progression of intestinal permeability changes and alpha-synuclein expression in a mouse model of Parkinson's disease. Mov Disord, 29:999-1009. 24898698 10.1002/mds.25736 PMC4050039

[9] Forsyth CB, Shannon KM, Kordower JH, Voigt RM, Shaikh M, Jaglin JA, et al. (2011). Increased intestinal permeability correlates with sigmoid mucosa alpha-synuclein staining and endotoxin exposure markers in early Parkinson's disease. PLoS One, 6:e28032. 22145021 10.1371/journal.pone.0028032 PMC3228722

[10] Dutta SK, Verma S, Jain V, Surapaneni BK, Vinayek R, Phillips L, et al. (2019). Parkinson’s Disease: The Emerging Role of Gut Dysbiosis, Antibiotics, Probiotics, and Fecal Microbiota Transplantation. Journal of Neurogastroenterology and Motility, 25:363-376. 31327219 10.5056/jnm19044 PMC6657920

[11] Marchiando AM, Graham WV, Turner JR (2010). Epithelial barriers in homeostasis and disease. Annu Rev Pathol, 5:119-144. 20078218 10.1146/annurev.pathol.4.110807.092135

[12] Suzuki T (2013). Regulation of intestinal epithelial permeability by tight junctions. Cell Mol Life Sci, 70:631-659. 22782113 10.1007/s00018-012-1070-x PMC11113843

[13] Camilleri M (2019). Leaky gut: mechanisms, measurement and clinical implications in humans. Gut, 68:1516-1526. 31076401 10.1136/gutjnl-2019-318427 PMC6790068

[14] Clairembault T, Leclair-Visonneau L, Coron E, Bourreille A, Le Dily S, Vavasseur F, et al. (2015). Structural alterations of the intestinal epithelial barrier in Parkinson's disease. Acta Neuropathol Commun, 3:12. 25775153 10.1186/s40478-015-0196-0 PMC4353469

[15] Saitou M, Furuse M, Sasaki H, Schulzke JD, Fromm M, Takano H, et al. (2000). Complex phenotype of mice lacking occludin, a component of tight junction strands. Mol Biol Cell, 11:4131-4142. 11102513 10.1091/mbc.11.12.4131 PMC15062

[16] Davies KN, King D, Billington D, Barrett JA (1996). Intestinal permeability and orocaecal transit time in elderly patients with Parkinson's disease. Postgrad Med J, 72:164-167. 8731708 10.1136/pgmj.72.845.164 PMC2398397

[17] Salat-Foix D, Tran K, Ranawaya R, Meddings J, Suchowersky O (2012). Increased intestinal permeability and Parkinson disease patients: chicken or egg? Can J Neurol Sci, 39:185-188. 22343151 10.1017/s0317167100013202

[18] Schoultz I, Keita AV (2020). The Intestinal Barrier and Current Techniques for the Assessment of Gut Permeability. Cells, 9: 1909 10.3390/cells9081909 PMC7463717 32824536

[19] Wallon C, Braaf Y, Wolving M, Olaison G, Soderholm JD (2005). Endoscopic biopsies in Ussing chambers evaluated for studies of macromolecular permeability in the human colon. Scand J Gastroenterol, 40:586-595. 16036512 10.1080/00365520510012235

[20] Unger MM, Spiegel J, Dillmann KU, Grundmann D, Philippeit H, Burmann J, et al. (2016). Short chain fatty acids and gut microbiota differ between patients with Parkinson's disease and age-matched controls. Parkinsonism Relat Disord, 32:66-72. 27591074 10.1016/j.parkreldis.2016.08.019

[21] Keshavarzian A, Green SJ, Engen PA, Voigt RM, Naqib A, Forsyth CB, et al. (2015). Colonic bacterial composition in Parkinson's disease. Movement Disorders, 30:1351-1360. 26179554 10.1002/mds.26307

[22] Dwyer Z, Chaiquin M, Landrigan J, Ayoub K, Shail P, Rocha J, et al. (2021). The impact of dextran sodium sulphate and probiotic pre-treatment in a murine model of Parkinson’s disease. Journal of Neuroinflammation, 18: 20 33422110 10.1186/s12974-020-02062-2 PMC7796536

[23] Laval L, Martin R, Natividad JN, Chain F, Miquel S, de Maredsous CD, et al. (2015). Lactobacillus rhamnosusCNCM I-3690 and the commensal bacteriumFaecalibacterium prausnitziiA2-165 exhibit similar protective effects to induced barrier hyper-permeability in mice. Gut Microbes, 6:1-9. 25517879 10.4161/19490976.2014.990784 PMC4615674

[24] Martín R, Miquel S, Chain F, Natividad JM, Jury J, Lu J, et al. (2015). Faecalibacterium prausnitzii prevents physiological damages in a chronic low-grade inflammation murine model. BMC Microbiology, 15. 25888448 10.1186/s12866-015-0400-1 PMC4391109

[25] Song S, Liu J, Zhang F, Hong JS (2020). Norepinephrine depleting toxin DSP-4 and LPS alter gut microbiota and induce neurotoxicity in alpha-synuclein mutant mice. Sci Rep, 10:15054. 32929122 10.1038/s41598-020-72202-4 PMC7490385

[26] Hasegawa S, Goto S, Tsuji H, Okuno T, Asahara T, Nomoto K, et al. (2015). Intestinal Dysbiosis and Lowered Serum Lipopolysaccharide-Binding Protein in Parkinson's Disease. PLoS One, 10:e0142164. 26539989 10.1371/journal.pone.0142164 PMC4634857

[27] Scheperjans F, Aho V, Pereira PAB, Koskinen K, Paulin L, Pekkonen E, et al. (2014). Gut microbiota are related to Parkinson's disease and clinical phenotype. Movement Disorders, 30:350-358. 25476529 10.1002/mds.26069

[28] Sun MF, Zhu YL, Zhou ZL, Jia XB, Xu YD, Yang Q, et al. (2018). Neuroprotective effects of fecal microbiota transplantation on MPTP-induced Parkinson's disease mice: Gut microbiota, glial reaction and TLR4/TNF-alpha signaling pathway. Brain Behav Immun, 70:48-60. 29471030 10.1016/j.bbi.2018.02.005

[29] Zhao Z, Ning J, Bao XQ, Shang M, Ma J, Li G, et al. (2021). Fecal microbiota transplantation protects rotenone-induced Parkinson's disease mice via suppressing inflammation mediated by the lipopolysaccharide-TLR4 signaling pathway through the microbiota-gut-brain axis. Microbiome, 9:226. 34784980 10.1186/s40168-021-01107-9 PMC8597301

[30] Kuai XY, Yao XH, Xu LJ, Zhou YQ, Zhang LP, Liu Y, et al. (2021). Evaluation of fecal microbiota transplantation in Parkinson's disease patients with constipation. Microb Cell Fact, 20:98. 33985520 10.1186/s12934-021-01589-0 PMC8120701

[31] Metta V, Leta V, Mrudula KR, Prashanth LK, Goyal V, Borgohain R, et al. (2021). Gastrointestinal dysfunction in Parkinson's disease: molecular pathology and implications of gut microbiome, probiotics, and fecal microbiota transplantation. J Neurol, in press. 10.1007/s00415-021-10567-w 33881598

[32] Klein RD, Shu Q, Cusumano ZT, Nagamatsu K, Gualberto NC, Lynch AJL, et al. (2018). Structure-Function Analysis of the Curli Accessory Protein CsgE Defines Surfaces Essential for Coordinating Amyloid Fiber Formation. mBio, 9. 10.1128/mBio.01349-18 PMC6050966 30018113

[33] Tursi SA, Tukel C (2018). Curli-Containing Enteric Biofilms Inside and Out: Matrix Composition, Immune Recognition, and Disease Implications. Microbiol Mol Biol Rev, 82. 10.1128/MMBR.00028-18 PMC6298610 30305312

[34] Sharma R, Young C, Neu J (2010). Molecular modulation of intestinal epithelial barrier: contribution of microbiota. J Biomed Biotechnol, 2010:305879. 20150966 10.1155/2010/305879 PMC2817557

[35] Koh A, De Vadder F, Kovatcheva-Datchary P, Backhed F (2016). From Dietary Fiber to Host Physiology: Short-Chain Fatty Acids as Key Bacterial Metabolites. Cell, 165:1332-1345. 27259147 10.1016/j.cell.2016.05.041

[36] Ploger S, Stumpff F, Penner GB, Schulzke JD, Gabel G, Martens H, et al. (2012). Microbial butyrate and its role for barrier function in the gastrointestinal tract. Ann N Y Acad Sci, 1258:52-59. 22731715 10.1111/j.1749-6632.2012.06553.x

[37] Venkatraman A, Ramakrishna BS, Pulimood AB, Patra S, Murthy S (2000). Increased permeability in dextran sulphate colitis in rats: time course of development and effect of butyrate. Scand J Gastroenterol, 35:1053-1059. 11099058 10.1080/003655200451171

[38] Yan H, Ajuwon KM (2017). Butyrate modifies intestinal barrier function in IPEC-J2 cells through a selective upregulation of tight junction proteins and activation of the Akt signaling pathway. PLoS One, 12:e0179586. 28654658 10.1371/journal.pone.0179586 PMC5487041

[39] Peng L, Li ZR, Green RS, Holzman IR, Lin J (2009). Butyrate enhances the intestinal barrier by facilitating tight junction assembly via activation of AMP-activated protein kinase in Caco-2 cell monolayers. J Nutr, 139:1619-1625. 19625695 10.3945/jn.109.104638 PMC2728689

[40] Tong LC, Wang Y, Wang ZB, Liu WY, Sun S, Li L, et al. (2016). Propionate Ameliorates Dextran Sodium Sulfate-Induced Colitis by Improving Intestinal Barrier Function and Reducing Inflammation and Oxidative Stress. Front Pharmacol, 7:253. 27574508 10.3389/fphar.2016.00253 PMC4983549

[41] Wang H-B, Wang P-Y, Wang X, Wan Y-L, Liu Y-C (2012). Butyrate Enhances Intestinal Epithelial Barrier Function via Up-Regulation of Tight Junction Protein Claudin-1 Transcription. Digestive Diseases and Sciences, 57:3126-3135. 22684624 10.1007/s10620-012-2259-4

[42] Hou Y, Li X, Liu C, Zhang M, Zhang X, Ge S, et al. (2021). Neuroprotective effects of short-chain fatty acids in MPTP induced mice model of Parkinson's disease. Exp Gerontol, 150:111376. 33905875 10.1016/j.exger.2021.111376

[43] Srivastav S, Neupane S, Bhurtel S, Katila N, Maharjan S, Choi H, et al. (2019). Probiotics mixture increases butyrate, and subsequently rescues the nigral dopaminergic neurons from MPTP and rotenone-induced neurotoxicity. J Nutr Biochem, 69:73-86. 31063918 10.1016/j.jnutbio.2019.03.021

[44] Maynard CL, Elson CO, Hatton RD, Weaver CT (2012). Reciprocal interactions of the intestinal microbiota and immune system. Nature, 489:231-241. 22972296 10.1038/nature11551 PMC4492337

[45] Shin C, Lim Y, Lim H, Ahn TB (2020). Plasma Short-Chain Fatty Acids in Patients With Parkinson's Disease. Mov Disord, 35:1021-1027. 32154946 10.1002/mds.28016

[46] Wallen ZD, Appah M, Dean MN, Sesler CL, Factor SA, Molho E, et al. (2020). Characterizing dysbiosis of gut microbiome in PD: evidence for overabundance of opportunistic pathogens. NPJ Parkinsons Dis, 6:11. 32566740 10.1038/s41531-020-0112-6 PMC7293233

[47] Li W, Wu X, Hu X, Wang T, Liang S, Duan Y, et al. (2017). Structural changes of gut microbiota in Parkinson's disease and its correlation with clinical features. Sci China Life Sci, 60:1223-1233. 28536926 10.1007/s11427-016-9001-4

[48] Capaldo CT, Nusrat A (2009). Cytokine regulation of tight junctions. Biochimica et Biophysica Acta (BBA) - Biomembranes, 1788:864-871. 18952050 10.1016/j.bbamem.2008.08.027 PMC2699410

[49] Li Y, Chen Y, Jiang L, Zhang J, Tong X, Chen D, et al. (2021). Intestinal Inflammation and Parkinson's Disease. Aging Dis, 12:2052-2068. 34881085 10.14336/AD.2021.0418 PMC8612622

[50] Villumsen M, Aznar S, Pakkenberg B, Jess T, Brudek T (2019). Inflammatory bowel disease increases the risk of Parkinson's disease: a Danish nationwide cohort study 1977-2014. Gut, 68:18-24. 29785965 10.1136/gutjnl-2017-315666

[51] Wang Q, Song S, Jiang L, Hon J-S (2021). Interplay among norepinephrine, NOX2, and neuroinflammation: key players in Parkinson’s disease and prime targets for therapies. Ageing and Neurodegenerative Diseases, 1: 6

[52] Houser MC, Tansey MG (2017). The gut-brain axis: is intestinal inflammation a silent driver of Parkinson’s disease pathogenesis? npj Parkinson's Disease, 3:3. 10.1038/s41531-016-0002-0 PMC5445611 28649603

[53] de Waal GM, Engelbrecht L, Davis T, de Villiers WJS, Kell DB, Pretorius E (2018). Correlative Light-Electron Microscopy detects lipopolysaccharide and its association with fibrin fibres in Parkinson's Disease, Alzheimer's Disease and Type 2 Diabetes Mellitus. Sci Rep, 8:16798. 30429533 10.1038/s41598-018-35009-y PMC6235901

[54] Perez-Pardo P, Dodiya HB, Engen PA, Forsyth CB, Huschens AM, Shaikh M, et al. (2019). Role of TLR4 in the gut-brain axis in Parkinson's disease: a translational study from men to mice. Gut, 68:829-843. 30554160 10.1136/gutjnl-2018-316844

[55] Bischoff SC, Barbara G, Buurman W, Ockhuizen T, Schulzke JD, Serino M, et al. (2014). Intestinal permeability--a new target for disease prevention and therapy. BMC Gastroenterol, 14:189. 25407511 10.1186/s12876-014-0189-7 PMC4253991

[56] Cryan JF, O'Riordan KJ, Cowan CSM, Sandhu KV, Bastiaanssen TFS, Boehme M, et al. (2019). The Microbiota-Gut-Brain Axis. Physiol Rev, 99:1877-2013. 31460832 10.1152/physrev.00018.2018

[57] Fukumoto S, Tatewaki M, Yamada T, Fujimiya M, Mantyh C, Voss M, et al. (2003). Short-chain fatty acids stimulate colonic transit via intraluminal 5-HT release in rats. Am J Physiol Regul Integr Comp Physiol, 284:R1269-1276. 12676748 10.1152/ajpregu.00442.2002

[58] Schwiertz A, Spiegel J, Dillmann U, Grundmann D, Bürmann J, Faßbender K, et al. (2018). Fecal markers of intestinal inflammation and intestinal permeability are elevated in Parkinson's disease. Parkinsonism & Related Disorders, 50:104-107. 29454662 10.1016/j.parkreldis.2018.02.022

[59] Mulak A, Koszewicz M, Panek-Jeziorna M, Koziorowska-Gawron E, Budrewicz S (2019). Fecal Calprotectin as a Marker of the Gut Immune System Activation Is Elevated in Parkinson's Disease. Front Neurosci, 13:992. 31611762 10.3389/fnins.2019.00992 PMC6776883

[60] Aho VTE, Houser MC, Pereira PAB, Chang J, Rudi K, Paulin L, et al. (2021). Relationships of gut microbiota, short-chain fatty acids, inflammation, and the gut barrier in Parkinson's disease. Mol Neurodegener, 16:6. 33557896 10.1186/s13024-021-00427-6 PMC7869249

[61] Seguella L, Sarnelli G, Esposito G (2020). Leaky gut, dysbiosis, and enteric glia activation: the trilogy behind the intestinal origin of Parkinson's disease. Neural Regen Res, 15:1037-1038. 31823880 10.4103/1673-5374.270308 PMC7034261

[62] Grubisic V, Gulbransen BD (2017). Enteric glia: the most alimentary of all glia. J Physiol, 595:557-570. 27106597 10.1113/JP271021 PMC5233670

[63] Savidge TC, Sofroniew MV, Neunlist M (2007). Starring roles for astroglia in barrier pathologies of gut and brain. Lab Invest, 87:731-736. 17607301 10.1038/labinvest.3700600

[64] Gulbransen BD, Sharkey KA (2012). Novel functional roles for enteric glia in the gastrointestinal tract. Nat Rev Gastroenterol Hepatol, 9:625-632. 22890111 10.1038/nrgastro.2012.138

[65] Van Landeghem L, Mahe MM, Teusan R, Leger J, Guisle I, Houlgatte R, et al. (2009). Regulation of intestinal epithelial cells transcriptome by enteric glial cells: impact on intestinal epithelial barrier functions. BMC Genomics, 10:507. 19883504 10.1186/1471-2164-10-507 PMC2778665

[66] Savidge TC, Newman P, Pothoulakis C, Ruhl A, Neunlist M, Bourreille A, et al. (2007). Enteric glia regulate intestinal barrier function and inflammation via release of S-nitrosoglutathione. Gastroenterology, 132:1344-1358. 17408650 10.1053/j.gastro.2007.01.051

[67] Aube AC, Cabarrocas J, Bauer J, Philippe D, Aubert P, Doulay F, et al. (2006). Changes in enteric neurone phenotype and intestinal functions in a transgenic mouse model of enteric glia disruption. Gut, 55:630-637. 16236773 10.1136/gut.2005.067595 PMC1856141

[68] Turco F, Sarnelli G, Cirillo C, Palumbo I, De Giorgi F, D'Alessandro A, et al. (2014). Enteroglial-derived S100B protein integrates bacteria-induced Toll-like receptor signalling in human enteric glial cells. Gut, 63:105-115. 23292665 10.1136/gutjnl-2012-302090

[69] Kabouridis PS, Lasrado R, McCallum S, Chng SH, Snippert HJ, Clevers H, et al. (2015). Microbiota controls the homeostasis of glial cells in the gut lamina propria. Neuron, 85:289-295. 25578362 10.1016/j.neuron.2014.12.037 PMC4306542

[70] Wang Q, Luo Y, Ray Chaudhuri K, Reynolds R, Tan EK, Pettersson S (2021). The role of gut dysbiosis in Parkinson's disease: mechanistic insights and therapeutic options. Brain, 144:2571-2593. 33856024 10.1093/brain/awab156

[71] Ruhl A, Franzke S, Collins SM, Stremmel W (2001). Interleukin-6 expression and regulation in rat enteric glial cells. Am J Physiol Gastrointest Liver Physiol, 280:G1163-1171. 11352809 10.1152/ajpgi.2001.280.6.G1163

[72] von Boyen GB, Steinkamp M, Geerling I, Reinshagen M, Schafer KH, Adler G, et al. (2006). Proinflammatory cytokines induce neurotrophic factor expression in enteric glia: a key to the regulation of epithelial apoptosis in Crohn's disease. Inflamm Bowel Dis, 12:346-354. 16670534 10.1097/01.MIB.0000219350.72483.44

[73] Meir M, Flemming S, Burkard N, Bergauer L, Metzger M, Germer C-T, et al. (2015). Glial cell line-derived neurotrophic factor promotes barrier maturation and wound healing in intestinal epithelial cells in vitro. American Journal of Physiology-Gastrointestinal and Liver Physiology, 309:G613-G624. 10.1152/ajpgi.00357.2014 26294673

[74] Bauman BD, Meng J, Zhang L, Louiselle A, Zheng E, Banerjee S, et al. (2017). Enteric glial-mediated enhancement of intestinal barrier integrity is compromised by morphine. Journal of Surgical Research, 219:214-221. 29078884 10.1016/j.jss.2017.05.099 PMC5708166

[75] von Boyen GBT, Steinkamp M, Geerling I, Reinshagen M, Sch??fer KH, Adler G, et al. (2006). Proinflammatory Cytokines Induce Neurotrophic Factor Expression in Enteric Glia. Inflammatory Bowel Diseases, 12:346-354. 16670534 10.1097/01.MIB.0000219350.72483.44

[76] Steinkamp M, Geerling I, Seufferlein T, von Boyen G, Egger B, Grossmann J, et al. (2003). Glial-derived neurotrophic factor regulates apoptosis in colonic epithelial cells. Gastroenterology, 124:1748-1757. 12806607 10.1016/s0016-5085(03)00404-9

[77] Devos D, Lebouvier T, Lardeux B, Biraud M, Rouaud T, Pouclet H, et al. (2013). Colonic inflammation in Parkinson's disease. Neurobiol Dis, 50:42-48. 23017648 10.1016/j.nbd.2012.09.007

[78] Van Landeghem L, Chevalier J, Mahe MM, Wedel T, Urvil P, Derkinderen P, et al. (2011). Enteric glia promote intestinal mucosal healing via activation of focal adhesion kinase and release of proEGF. Am J Physiol Gastrointest Liver Physiol, 300:G976-987. 21350188 10.1152/ajpgi.00427.2010 PMC3119120

[79] Clairembault T, Leclair-Visonneau L, Neunlist M, Derkinderen P (2015). Enteric glial cells: new players in Parkinson's disease? Mov Disord, 30:494-498. 25100667 10.1002/mds.25979

[80] Clairembault T, Kamphuis W, Leclair-Visonneau L, Rolli-Derkinderen M, Coron E, Neunlist M, et al. (2014). Enteric GFAP expression and phosphorylation in Parkinson's disease. J Neurochem, 130:805-815. 24749759 10.1111/jnc.12742

[81] Lema Tome CM, Tyson T, Rey NL, Grathwohl S, Britschgi M, Brundin P (2013). Inflammation and alpha-synuclein's prion-like behavior in Parkinson's disease--is there a link? Mol Neurobiol, 47:561-574. 22544647 10.1007/s12035-012-8267-8 PMC3589652

[82] Shannon KM, Keshavarzian A, Mutlu E, Dodiya HB, Daian D, Jaglin JA, et al. (2012). Alpha-synuclein in colonic submucosa in early untreated Parkinson's disease. Mov Disord, 27:709-715. 21766334 10.1002/mds.23838

